# Rare Earth Element Adsorption from Water Using Alkali-Activated Waste Fly Ash

**DOI:** 10.3390/ma18030699

**Published:** 2025-02-05

**Authors:** Tijana Radojičić, Katarina Trivunac, Marija Vukčević, Marina Maletić, Nataša Palić, Ivona Janković-Častvan, Aleksandra Perić Grujić

**Affiliations:** 1 Faculty of Technology and Metallurgy, University of Belgrade, Karnegijeva 4, 11120 Belgrade, Serbia; radojicic.tijana96@gmail.com (T.R.); marijab@tmf.bg.ac.rs (M.V.); alexp@tmf.bg.ac.rs (A.P.G.); 2 Innovation Center of Faculty of Technology and Metallurgy, Karnegijeva 4, 11120 Belgrade, Serbia; mvukasinovic@tmf.bg.ac.rs (M.M.); nkaric@tmf.bg.ac.rs (N.P.); icastvan@tmf.bg.ac.rs (I.J.-Č.)

**Keywords:** rare earth elements, amorphous gel, fly ash, wood ash, adsorption, wastewater

## Abstract

As new technologies are developed, the demand for rare earth elements (REEs) has increased, despite limited awareness of their significant impact on people and the environment. In this study, waste fly ash was used as a precursor to synthesize inorganic aluminosilicate polymers by adding an activator to the alumina and silica compounds of the ash. Due to their structure and adsorption potential, their application for the removal of selected REEs (Gd^3+^, Y^3+^, and Sc^3+^) from water has been investigated. A decrease in the intensity of the quartz peak at 2θ of 26.6° in the XRD spectrum and the disappearance of the albite and mullite peaks due to dissolution during alkaline activation in both modified samples were observed. The appearance of a peaks at 2θ of 29.3° and 39.3° corresponding to calcite in the modified sample indicates the presence of wood ash. A shifting of the band in the DRIFT spectrum to 1030 cm^−1^ on the spectra of modified samples corresponds to the vibrations of Al-O and Si-O bonds and the formation of a polymeric network structure (Si-O-Si or Si-O-Al). According to pH_PZC_ values, thermodynamic and kinetic parameters, and chemical composition, the presumed mechanism of REE adsorption is chemisorption and ion exchange. The highest adsorption efficiencies (up to 95%) for all examined REEs in both single and mixed REE solutions were obtained from an alkali-activated mixture of fly ash and wood ash. The results of this research are significant for expanding knowledge about the removal of REEs from the environment, the reduction of waste ash by their modification, and their potential subsequent use in construction as additives.

## 1. Introduction

In recent years, awareness and interest in rare earth elements (REEs) have been growing due to their presence in various high-tech processes in many branches of industry. These elements consist of yttrium (Y), scandium (Sc), and 15 lanthanides. The most general division of REEs is into light (La, Ce, Pr, Nd, Pm, Sm, Eu, and Gd) and heavy (Y, Tb, Dz, Ho, Er, Tm, Zb, and Lu) elements. Scandium and yttrium do not belong to the lanthanide family, but are included because they are found in the same mineral deposits as the lanthanides and also have similar chemical properties [1]. Rare earth elements are an integral part of modern technology, the electronics industry, and the nuclear and energy sectors, from mobile phones and televisions to batteries, lasers, superconductors, super-magnets, LED bulbs, and hybrid and electric cars and windmills [2]. In addition, their use is expanding to agriculture and into medicine, especially using gadolinium as a contrast agent in magnetic resonance medicine [3]. REEs have become important to the world of technology because of their unique magnetic, phosphorescent, and catalytic properties. In the natural environment, rare earth elements do not occur as individual natural metals, such as gold or silver, but occur together in many ores or minerals as secondary or major constituents. With the development of new technologies, the increased demand for them leads to extensive exploitation and reductions in their natural resources. The extraction of rare earth elements is a complex, multi-step process that varies depending on the rare earth elements and the raw materials used. Due to the possible low concentration of REEs, their separation among the abundant contaminating components is difficult; finding a method that is efficient and green is a big modern challenge [4]. The most common techniques that can be used are leaching [5], e.g., dissolving the ore with strong acids (sulfuric or hydrochloric acid) and extracting the REEs; extraction with an organic solvent; ion exchange separation [6]; the precipitation of certain REEs [7]; the magnetic separation of rare earth minerals with paramagnetic properties; charge-based separation by electrodialysis; and membrane extraction for the separation of REEs from aqueous and gaseous phases. The extraction of rare earth elements produces a significant amount of toxic and radioactive materials that can cause contamination. The future of REEs will be greatly influenced by the consequences of the exploitation and processing of REEs on the environment and human health. For economic and ecological reasons, more attention is paid to secondary sources of these strategic elements, but also to efficient ways of recycling them [8]. Recently, much attention has been focused on other potential sources of rare earth elements such as mining waste and coal combustion by-products [2]. Fly ash, which is considered a waste, is a possible source of many elements, including REEs, because their concentrations are equal to or higher than those found in conventional ores [5,9,10]. Intensive research is currently underway to develop more efficient technologies for the recovery of rare earth elements from various alternative sources (industrial and electronic waste, red mud, waste phosphors, fluorescent lamps, and batteries), but without consequences for the ecosystem [8,11,12,13,14]. The increased presence of rare earth elements in various products for industrial or human use inevitably leads to increased concentrations in the environment due to improper waste disposal, and through human excretions that can be found in municipal wastewater [15]. Water treatment plants do not provide for the removal of REEs, so they end up in the environment where, in addition to water pollution, they can bioaccumulate in living organisms and accumulate in sediments or soil [16]. This problem has been recognized by the scientific community and has stimulated research on the presence of anthropogenic REEs in various mediums, their toxicity, and their impact on human health, as well as ways to reduce them [17,18,19]. The adsorption process is considered a potential alternative to other methods for the separation and removal of rare earth metals in wastewater treatment processes. The process is recognized as an efficient, economically viable, technically feasible, and widely used approach [20]. Although the development of the adsorbents used is the most important part of the development of this method, for the efficiency of the whole process it is very important to optimize all the working parameters. To reduce additional environmental pollution and the consumption of large amounts of chemicals, researchers are focusing on using natural and even more waste materials that can be given new utility value by modifying and applying them as sorbents for various pollutants. One such material is fly ash, which remains as a by-product after coal combustion. Some of this waste is used in construction and for roads, but most of it remains in landfills and pollutes water, air, and soil. Therefore, this material has been subjected to various methods of chemical, mechanical, and thermal modification and has proven to be very successful in removing pharmaceuticals, dyes, and heavy metals from wastewater [21,22]. The most commonly used precursor for obtaining geopolymers with an aluminosilicate gelatinous structure is metakaolin, although fly ash, bottom ash, red mud, zeolites, etc., can also be used. Metakaolin-based materials showed a high adsorption capacity for tetracycline (445 mg/g) and significant capacity for the heavy metals lead (246.4 mg/g) and zinc (74.5 mg/g). The lowest result was obtained for methylene blue (26.4 mg/g). Slightly different values were obtained during experiments on fly ash-based materials, where adsorption capacity values of 84 mg/g for methylene blue, 83.2 mg/g for lead, and 13.42 mg/g for zinc were obtained. Salam et al. used peanut shells, fly ash, and natural zeolite to remove heavy metals (copper and zinc) from wastewater [23]. The effectiveness of geopolymers based on metakaolin, or obtained by mixing metakaolin precursors and fly ash, in the removal of lead and cadmium ions was investigated by several authors [24,25]. The maximum adsorption capacity of geopolymers prepared using a mixture of coal fly ash and metakaolin for the removal of Pb^2+^ and Cd^2+^ was 164.1 and 78.2 mg/g, respectively. In another study, where the precursor for obtaining the geopolymer was metakaolin, the adsorption capacity had a value of 43.7 mg/g. Fly ash, in addition to being used as a source for the extraction of rare elements, can potentially serve as an effective adsorbent after modification.

This study aimed to evaluate the possibility of using alkaline-activated fly ash as an adsorbent for the separation of selected rare earth elements from an acidic aqueous solution, since the application of these inorganic polymeric materials for the removal of gadolinium, yttrium, and scandium has not been reported in the literature. Thus, in this work, alkaline activation of raw fly ash and fly ash with the addition of 20 percent wood ash was performed. During synthesis, aluminosilicate gelous structures are formed [26], improving the material’s ability to bind pollutants in the adsorption process. The samples were characterized using diffuse reflectance infrared Fourier transform (DRIFT), scanning electron microscopy (SEM), and X-ray diffraction (XRD). Determination of the point of zero charge (pH_PZC_) was carried out. The influence of the initial pH value and initial concentration, as well as the influence of the contact time and temperature on adsorption, were also examined. The experimental data were processed using Langmuir and Freundlich isotherm models, intraparticular diffusion, and pseudo-first and second-order kinetic models, and the thermodynamic parameters of the process were calculated.

## 2. Materials and Methods

### 2.1. Material

The fly ash (FA) used in this study was obtained from the coal combustion (Thermal power plant Nikola Tesla B, Obrenovac, Serbia), while wood ash (20%) was obtained from a burning process in individual fireplaces. The chemicals used for material modification and adsorption experiments were sodium hydroxide (pellets, ≥97%, CAS No: 1310-73-2), sodium silicate (>99%, CAS No: 1344-09-8), sodium chloride (≥99.5%, CAS No: 7647-14-5), and hydrochloric acid (37%, CAS No: 7647-01-0), and were supplied by Sigma-Aldrich (St. Louis, MO, USA).

The stock solutions (1.00 g L^−1^) of the elements for the adsorption test were prepared from the salts Y^3+^ (Y(NO_₃_)_₃_·6H_₂_O, 99.8%, CAS No: 13494-98-9), Sc^3+^ (solution of Sc(NO_₃_)_₃_·H_₂_O, 99.9%, CAS No: 107552-14-7), and Gd^3+^ (solution of Gd(NO_₃_)_₃_·6H_₂_O, 99.99%, CAS No: 19598-90-4), also purchased from Sigma-Aldrich (St. Louis, MO, USA).

### 2.2. Material Sample Preparation

Fly ash was alkali-activated using 6 M NaOH and sodium silicate (molar modulus SiO_2_/Na_2_O was 3.1) solution in the volume ratio of 1.5, while the ratio between FA and the liquid phase of the alkali activator was 1:1. In this way, the alkali-activated sample FAmod_1_ was obtained. For preparing sample FAmod_2_, FA (80%) was mixed with wood ash (20%), obtained from a burning process in individual fireplaces. The mixture of fly and wood ash used a sodium hydroxide/sodium silicate solution. For alkali activation, the mixture of ashes was homogenized with a sodium hydroxide/sodium silicate solution, poured into molds, covered, and left at room temperature for 24 h. After that, the mixture was kept for 48 h in the oven at 60 °C, and then left for 28 days at room temperature in controlled conditions. The unmodified and modified samples were grounded, sieved (sieve size 355 µm), and washed with distilled water to a neutral pH.

### 2.3. Material Characterization

Field emission scanning electron microscopy (FESEM Mira3, Tescan Orsay Holding, Brno, Czech Republic) was used for the analysis of fly ash and modified fly ash samples sputtered with gold.

X-ray diffraction (XRD) patterns for all the materials under investigation were obtained using an PROTO AXRD Benchtop Powder Diffractometer (Proto Manufacturing Ltd. LaSalle, ON, Canada), which employs CuKα1,2 radiation. The XRD data were collected over a 2θ range of 15–70°, utilizing a continuous scan mode. The scanning was conducted with a step size of 0.015° and at a rate of 5° per minute.

Diffuse reflectance infrared Fourier transform spectroscopy (DRIFT, Perkin-Elmer FTIR spectrometer Spectrum Two, Waltham, MA, USA) was used to determine the type of surface functional groups on the examined materials. The spectra were recorded in the region from 4000 to 500 cm^−1^ with a resolution of 4 cm^−1^.

For determination of the point of zero charge (pH_PZC_), 0.06 g of examined material was immersed in 20 cm^3^ of 0.01 M NaCl solutions with the initial pH (pH_i_) ranging from pH 2 to pH 12 (with a step of 2), using 0.1 M HCl or 0.1 M NaOH for pH adjustment. After introducing nitrogen, cuvettes were sealed and shaken for 48 h at room temperature. The final pH (pH_f_) of solutions was measured, and the dependencies pH_f_–pH_i_ were plotted to determine pH_PZC_ [27].

### 2.4. Adsorption Experiments

Unmodified fly ash and differently modified samples were used for the adsorption of rare earth elements, scandium, yttrium, and gadolinium, from aqueous solutions. The adsorption of selected REEs from the single and multi-element aqueous solution was carried out in a batch system in glass beakers with constant shaking (150 rpm) at room temperature. For these experiments, 0.05 g of unmodified and modified fly ash samples was immersed in 20 cm^3^ of REE aqueous solution with an initial concentration of 10 mg/dm^3^ per element. The adsorption of REEs from a single-element solution was optimized through the examination of the influence that contact time and the initial pH and concentration have on adsorption. The influence of the initial pH of the REE aqueous solution on adsorption was investigated by adjusting the starting pH to 2.5, 3, 3.5, and 4, with an accuracy of ±0.01, using a diluted HCl and NaOH solution. According to the obtained results, as well as the activities and speciation of ions present in the solution (obtained by Visual Minteq 3.1 software) and the adsorbent pHpzc, the optimal initial pH value of the adsorbate solution was selected and used in the following experiments. The influence of contact time was assessed by the adsorption of REEs onto 0.05 g of adsorbent, from 20 cm^3^ of initial concentration 15 mg/dm^3^. Samples were taken at certain time intervals, namely 10, 30, 60, 120, 180, and 1440 min, and the concentration of selected REEs in all adsorption experiments was measured by Inductively Coupled Plasma Optical Emission spectroscopy (ICP-OES, Agilent, Santa Clara, CA, USA). Also, the influence of the initial REE concentration (7.5, 10, 15, 20, and 25 mg/dm^3^) on the adsorption capacity of fly ash samples was examined. Experimental data were analyzed using the following theoretical models: pseudo-first-order (Equation (1)) [28] and pseudo-second-order (Equation (2)) [29] kinetic models; intraparticle diffusion (Equation (3)) [30] for adsorption kinetics; and the Langmuir (Equation (4)) [31] and Freundlich (Equation (5)) [32] isotherm models for equilibrium data interpretation.

(1) $$q_{t}=q_{e}\cdot \left(1-e^{-k_{1}t}\right)$$

(2) $$q_{t}=q_{e}-{\left(\frac{1}{q_{e}}+k_{2}\cdot t\right)}^{-1}$$

(3) $$q_{t}=k_{id}\cdot t^{\frac{1}{2}}+C$$

(4) $$q_{e}=\frac{Q_{0}\cdot b\cdot C_{e}}{1+b\cdot C_{e}}$$

(5) qe=Kf·Ce1n

where *q_e_* and *q_t_* (mg/g) are the amounts of REEs adsorbed at equilibrium and at time *t* (min), respectively; *C_e_* is the equilibrium REE concentration (mg/dm^3^); *Q*_0_ is the amount of adsorbate adsorbed per unit mass of adsorbent required for monolayer coverage of the surface (mg/g); *b* is a constant related to the heat of adsorption (dm^3^/mg); *K*_f_ (mg g^−1^(mg dm^−3^)^−1/*n*^) is a Freundlich constant, related to the adsorption capacity; 1/*n* is the heterogeneity factor; *k*_1_ (1/min) and *k*_2_ (g/(mg min)) are the pseudo-first-order and pseudo-second-order rate constants, *k_id_* (mg/g min^1/2^) is the intraparticle diffusion rate constant that can be evaluated from the slope of the linear plot of *q_t_* versus *t*^1/2^, and the constant *C* is the intercept.

To examine the influence of temperature on adsorption capacities, adsorption of selected REE ions from 20 cm^3^ of solution (initial REE concentration of 10 mg/dm^3^) onto fly ash samples (0.05 g) was performed at different temperatures: 298.15, 308.15, and 318.15 K. From the experimental data, and using Equation (6), thermodynamic parameters ∆*H* and ∆*S* were obtained from the slopes and intercepts of *lnK*–1/*T* dependencies, respectively [33].

(6) $$\ln K=\frac{\Delta S}{R}-\frac{\Delta H}{RT}$$

The values of ∆*G* were calculated from the corresponding values of ∆*H* and ∆*S* following Equation (7):

(7) ΔG=ΔH−TΔS

Competitive adsorption on the modified fly ash samples was examined from the mixture of selected REE ions to imitate the conditions in real systems. For that purpose, 0.05 g of material was immersed in 20 cm^3^ of metal ion solution (initial concentration per REE species of 10 mg/dm^3^), and adsorption was conducted in a batch system at room temperature with constant shaking.

## 3. Results and Discussion

### 3.1. Material Characterization

**Figure 1:**
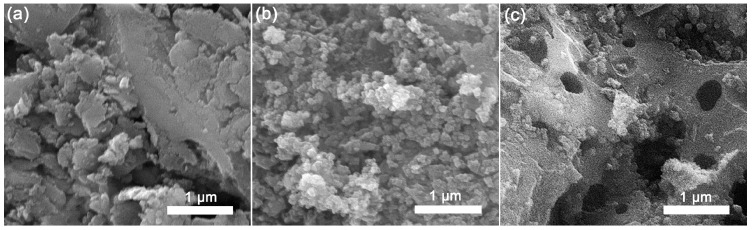
SEM photographs of (**a**) FA, (**b**) FAmod_1_, and (**c**) FAmod_2_.

Scanning electron microscopy revealed that alkali activation of fly ash (Figure 1a) led to agglomerated small particles forming on the surface of sample FAmod_1_ (Figure 1b), which resulted in increased roughness and geometrical surface area. On the other hand, the morphology of sample FAmod_2_, obtained by alkali activation of a mixture of fly and wood ash (Figure 1c), was characterized by large pores and cracks.

The chemical composition of the unmodified and modified fly ash samples was determined by EDS analysis. The results are shown in Table 1. The raw fly ash sample consists predominantly of oxygen (O), aluminum (Al), and silicon (Si), with small percentages of carbon (C), potassium (K), magnesium (Mg), and sodium (Na). After modification, the increase in sodium (Na) content was noticeable in both modified samples. The higher sodium content may be beneficial due to the reactivity of this ion and its easy exchangeability, which may contribute to the adsorption process of different metal ions through the ion exchange mechanism. A slight increase in calcium (Ca) content is observed in the FAmod_2_ sample. This is the result of adding wood ash, which contains a higher percentage of calcium.

**Figure 2:**
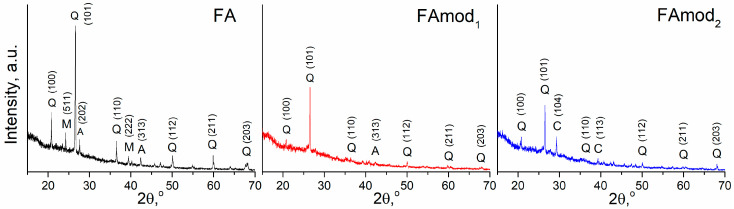
XRD spectra with Miller indices of unmodified and modified fly ash samples.

Unmodified fly ash and modified samples, FAmod_1_ and FAmod_2_, are characterized by an amorphous and semi-crystalline structure. The confirmation of the presence of an amorphous gelous phase is visible on XRD spectra (Figure 2) as a characteristic “hump” in the 2θ range from 20 to 40°. The crystalline phase contains quartz (Q), mullite (M), albite (A), and calcite (C) (originating from calcium in wood ash [34]) in the structure of FAmod_2_. The most intense quartz peak at 2θ of 26.6° decreases due to the applied modification, while the peak corresponding to the presence of albite (2θ = 27.5°) almost disappears. Also, the peaks for mullite, observed on the XRD spectra of FA, are not present on the XRD spectra of modified samples due to mullite dissolving during alkali activation.

**Figure 3:**
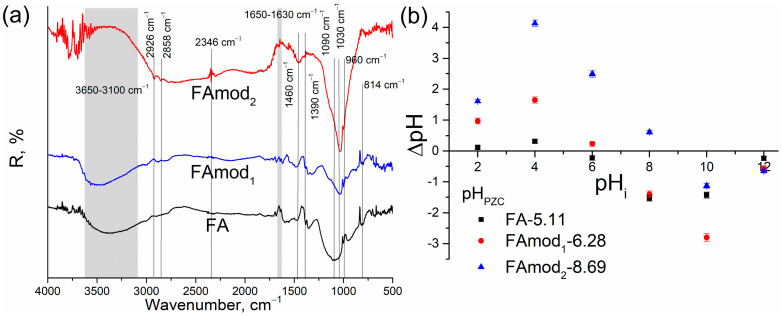
DRIFT spectra (**a**) and pH_PZC_ (**b**) of unmodified and modified fly ash samples.

Diffuse reflectance infrared Fourier transform (DRIFT) spectroscopy was used to determine the functional groups of all samples (Figure 3a). The wide bands in the region 3650–3100 cm^−1^ observed in the DRIFT spectra of FA and FAmod_1_ originate from surface hydroxyl groups. For all examined samples, the low-intensity peaks observed in the range 1650–1630 cm^−1^ can be assigned to the H-O-H bending vibration in the adsorbed water [35,36,37], while the peaks at around 1460 and 1390 cm^−1^ are typical for C-O stretching vibrations and can be attributed to calcium carbonate [35]. For FA, a wide band at approximately 1090 cm^−1^ was associated with asymmetric stretching vibrations of Si-O-Si and Al-O-Si [38], while the bands at 960 cm^−1^ and 814 cm^−1^ were attributed to the Si-O asymmetric stretching vibrations [39]. Vibrations associated with symmetric and asymmetric stretches of C-H in the methyl and methylene groups were found on the spectrum of FAmod_2_ at wavelengths 2858 cm^−1^ and 2926 cm^−1^, respectively. Also, the low-intensity peak at around 2346 cm^−1^ on the FAmod_2_ spectrum corresponds to the infrared band position of HCO_3_^−^ ions [40,41]. These peaks observed only on the spectra of FAmod_2_ are the consequence of the applied modification. The influence of alkali activation on surface properties is visible on the DRIFT spectra as a shifting of the band at 1090 cm^−1^ (spectra of FA) to the lower wavenumbers, i.e., to around 1030 cm^−1^ on the spectra of FAmod_1_ and FAmod_2_. This band at around 1033 cm^−1^, characteristic for the alkali-activated samples, corresponds to the vibrations of Al-O and Si-O bonds [42] and the formation of a polymeric gelous network structure (Si-O-Si or Si-O-Al) as a result of applied modification.

The applied alkali activation can affect the surface charge of examined samples and their adsorption properties, and therefore the point of zero charge (PZC) was determined from the ΔpH–pH_i_ dependence (Figure 3b). It was observed that the alkali activation of fly ash and the mixture of fly and wood ash increase the pH_PZC_ value, i.e., the pH value of the solution at which the observed charge of the material surface is zero. In solutions with a pH value lower than pH_PZC_, the functional groups are protonated and the surface of the adsorbent is positively charged. When the pH value of the solution is higher than pH_PZC_, the functional groups are deprotonated and the material has a negatively charged surface suitable for binding positive particles [22]. The obtained pH_PZC_ values are given in Figure 3b.

### 3.2. Adsorption Experiments

**Figure 4:**
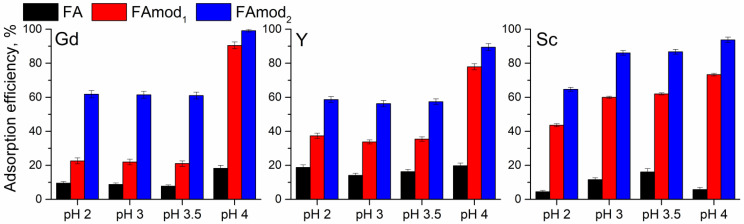
The influence of initial pH of rare earth metal ion solution on adsorption efficiency of FA, FAmod_1_, and FAmod_2_.

The initial pH of a rare earth element aqueous solution can be a dominant factor that affects the adsorption efficiency of unmodified and modified fly ash samples since the ionization state of the functional groups of the adsorbent surface is influenced by pH, as well as speciation of metal ions in the solution. Therefore, the influence of initial pH on adsorption efficiency is presented in Figure 4.

**Figure 5:**
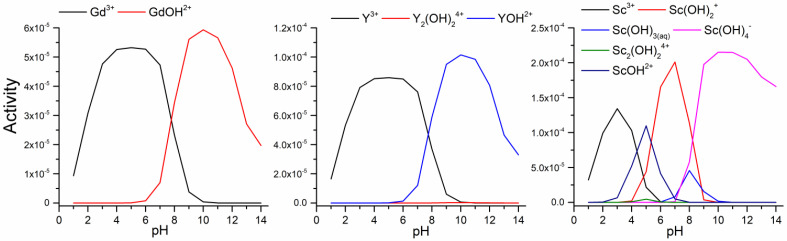
Speciation of gadolinium, yttrium, and scandium ions.

According to the speciation of rare earth metals (Figure 5), the examination was performed in a pH range from 2 to 4 to avoid the formation of hydroxides and precipitation. In this range, the dominant species of selected rare earth metals are Gd^3+^, Y^3+^, Sc^3+^, and ScOH^2+^, whose ratio increases above pH 3. According to the pH_PZC_ values, in the examined pH range, the surfaces of all samples are protonated and positively charged, and it can be assumed that a positively charged surface will repel the positively charged species. On the contrary, the obtained results (Figure 4) showed that adsorption efficiency for all rare earth elements increases with pH_PZC_ value, so the sample FAmod_2_ with a pH_PZC_ of 8.69 showed the highest adsorption efficiency for all tested initial pH values. The efficiency of all examined samples for the adsorption of Gd and Y ions in the pH range of 2–3.5 is not influenced by the initial pH value, while for pH 4 the adsorption efficiency is increased. On the other hand, the adsorption of scandium species onto the tested samples is influenced by the initial pH, and increases with the pH value, except for adsorption onto FA. To avoid potential hydroxide formation, particularly in the case of scandium, the initial pH value was adjusted and maintained around pH 3.5.

**Figure 6:**
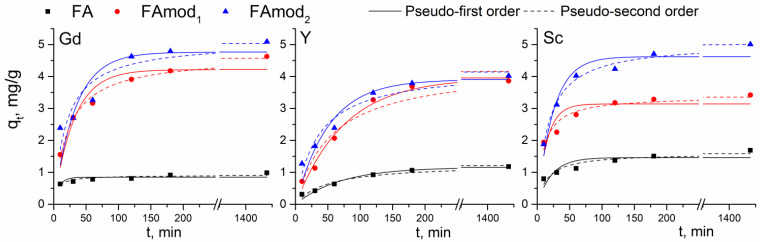
The influence of contact time on adsorption capacities and fitting experimental data with pseudo-first- and pseudo-second-order kinetic models.

The influence of contact time on the adsorption capacities of examined samples is given in Figure 6. The adsorption of REEs onto examined fly ash samples is a relatively fast process, as approximately 80–95% of the equilibrium adsorption capacity was achieved within the first hour of adsorption. The lowest equilibrium adsorption capacity for all three elements was obtained for the adsorption onto the unmodified FA sample, while FAmod_2_ showed the highest adsorption capacities.

Experimental data were analyzed using pseudo-first- and pseudo-second-order kinetic models, and the obtained kinetic parameters are summarized in Table 2. According to the correlation coefficients (*R^2^*), adsorption of REEs onto tested samples follows the pseudo-second-order kinetic model, except for the adsorption of yttrium onto FAmod_1_. On the other hand, the comparison of calculated (*q_e_*_,cal_) and experimentally obtained (*q_e_*_,exp_) values of equilibrium adsorption capacities indicates that adsorption of gadolinium and scandium on all examined samples follows the pseudo-second order kinetic model, while the adsorption of yttrium onto FA and FAmod_1_ and FAmod_2_ follows the pseudo-first-order kinetic model.

**Figure 7:**
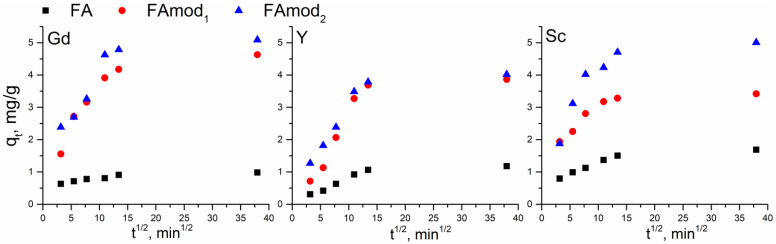
Intraparticle diffusion model for adsorption of gadolinium, yttrium, and scandium onto FA, FAmod_1,_ and FAmod_2_.

The intraparticle diffusion model was employed to identify the process that most significantly affects the adsorption rate and examine the REE adsorption mechanism onto fly ash samples. The intraparticle diffusion plots are given in Figure 7 and the intraparticle diffusion rate constant (*k_id_*) and constant *C* are summarized in Table 3.

The multi-linear intraparticle diffusion plots obtained for all examined samples suggest that the rate of adsorption of REEs could be affected by intraparticle diffusion, although it is not the only rate-controlling process. As can be seen from Figure 7, the adsorption of all REEs onto FA, as well as the adsorption of Y onto all examined samples, occurs through two consecutive steps, external mass transfer and slow equilibrium adsorption. On the other hand, adsorption of gadolinium and scandium onto FAmod_1_ and FAmod_2_ occurs through external mass transfer, diffusion through the liquid film surrounding the adsorbent and adsorption within the particle and on the external surface, and slow equilibrium adsorption.

**Figure 8:**
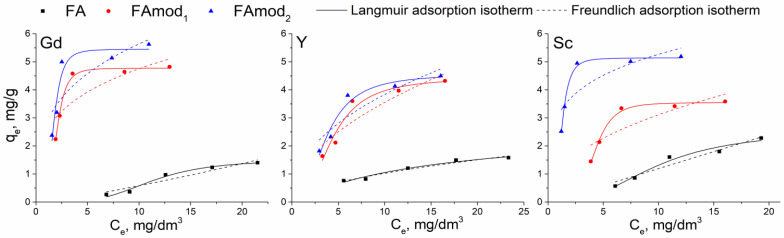
The influence of the initial concentration on adsorption capacities and fitting experimental data with Langmuir and Freundlich adsorption isotherm models.

The influence of the initial concentration of REE solution on adsorption is presented as the dependence of equilibrium adsorption capacity from equilibrium concentration (Figure 8). With the increase in the initial concentration, the adsorption capacities increase until reaching the characteristic plot that indicates the surface saturation. This plot is visible for the adsorption onto modified samples. At the same time, a slight constant increase in the adsorption capacity with the initial concentration was observed for unmodified FA, which showed the lowest adsorption capacities. Modified samples showed the highest capacities for gadolinium adsorption, while applied modification with wood ash had the strongest influence on scandium adsorption.

The obtained experimental data were analyzed with Langmuir and Freundlich adsorption isotherms, and the obtained isotherm parameters and the correlation coefficients (*R*^2^) are summarized in Table 4. According to the correlation coefficient values, the equilibrium adsorption of REE species onto unmodified and modified fly ash samples is described well by the Langmuir isotherm model, indicating the homogenous distribution of surface active sites and monolayer adsorption. The homogeneous distribution of active sites is also indicated by the obtained values of the surface heterogeneity factor, 1/*n* (Table 4), calculated based on the Freundlich model. The values of 1/*n* are less than one for the adsorption of the tested REE on the modified samples and the adsorption of yttrium on the FA sample.

The influence of surrounding temperature on REE adsorption was examined by performing adsorption experiments at three different temperatures (298.15, 308.15, and 318.15 K). Thermodynamic parameters and adsorption capacities at various temperatures are given in Table 5. Negative values of ∆*G* indicate that only adsorption of Sc onto FAmod_1_ and that of Y (at 308.15 and 318.15 K), Sc, and Gd onto FAmod_2_ are spontaneous processes. According to the positive values of enthalpy change, the process of REE adsorption onto unmodified and modified fly ash samples is endothermic, and the adsorption capacities increase with temperature. The increase in surrounding temperature leads to the higher mobility of REE species and better adsorption due to easier diffusion through the adsorbent boundary layer and porous surface. The obtained entropy values are positive, which means that entropy increases as a cause of adsorption due to the redistribution of energy between the REE ions and adsorbent [19].

The ∆*G* and ∆*H* values provide insight into whether the adsorption process is chemisorption or physisorption. Typically, physical adsorption is characterized by an enthalpy change between 2 and 21 kJ/mol and a Gibbs free energy between −20 and 0 kJ/mol. Additionally, chemical adsorption exhibits enthalpy values ranging from 80 to 200 kJ/mol and Gibbs free energy between −400 and −80 kJ/mol [43,44]. As indicated by the Gibbs free energy values in Table 5, spontaneous processes of the adsorption of scandium on FAmod_1_ and all REEs on FAmod_2_ appear to be physisorption. However, the enthalpy values, which range from 24.25 to 194.8 kJ/mol, suggest that the adsorption of REEs onto fly ash samples can be considered chemisorption. According to the pH_PZC_ values, the surface of fly ash samples is positively charged in the adsorbate solution (pH maintained at 3.5) during adsorption. This indicates that the adsorption of REEs takes place against the repulsive electrostatic forces, which is a strong argument in favor of chemisorption.

**Figure 9:**
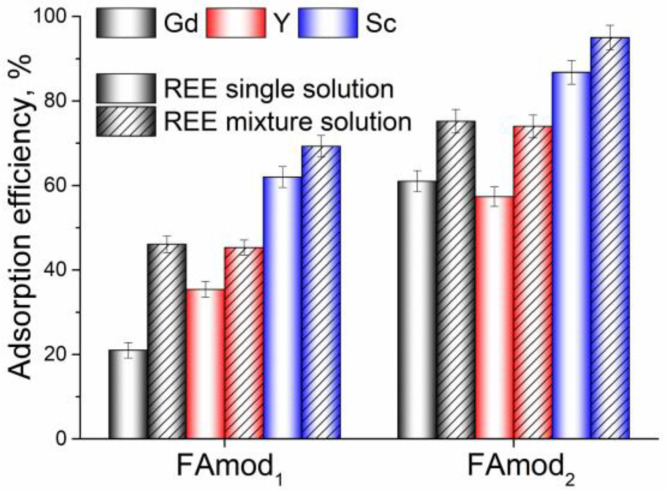
The adsorption efficiency of FAmod_1_ and FAmod_2_ for the removal of REEs from a single-element and mixed-element solution.

In order to examine the competition and mutual influence of REE species in solution, and to reproduce the conditions in real systems, adsorption of REEs was performed from a single-element solution and a solution of a mixture of REEs. As the previous experiments showed that applied modifications positively affect the adsorption properties of fly ash, modified samples were used as adsorbents for competitive adsorption. The efficiency of modified fly ash samples to adsorb REEs from a single-element and mixed-element solution is presented in Figure 9.

Sample FAmod_2_ showed higher adsorption efficiencies for all examined REEs in both single and mixed REE solutions. It can be noted that the presence of other REE species favorably affects adsorption, as the modified samples showed higher adsorption efficiency from the mixed REE solution. The higher adsorption efficiency from the mixed REE solution may result from the higher number of ion species present in the solution and the higher concentration gradient, which is a driving force for the adsorption. The synergistic effect observed between REE species during adsorption from a mixed solution indicates that these ions may either be adsorbed to distinct active sites or that the adsorption process occurs through different mechanisms.

Values of the adsorption capacity of different adsorbents for investigated rare earth elements found in the literature along with the results from this study are presented in Table 6. Although the comparison is limited by the different operating parameters of the adsorption process, it can be concluded that the obtained results for adsorbent tested in this study are comparable to or better than the presented data especially in the acidic pH range. The advantage of this adsorbent is the use of waste material that exists in large quantities in the pile, unlike the mentioned pitaya peels or coconut shells. Also, additional chemicals in the material preparation process are available, cheap, and used in small quantities. At the end of the process after the removal of rare elements, the spent adsorbent can be used as additives for the building materials [38,39].

## 4. Conclusions

In this study, the efficiency of alkaline-activated fly ash as an adsorbent for the separation of selected rare earth elements from an acidic aqueous solution has been evaluated. The decrease in the intensity of the quartz peak at 2θ of 26.6° and the disappearance of peaks corresponding to the presence of albite and mullite in the XRD spectra of the modified FAmod_1_ and FAmod_2_ samples indicate changes in the crystallinity of the material. In the FAmod_2_ sample, a crystalline phase of calcite (originating from calcium in wood ash) is visible, which influenced the stabilization of fly ash. The most significant change in the DRIFT spectra is a slight shift in the band at 1033 cm^−1^, which corresponds to vibrations of Al-O and Si-O bonds, i.e., the formation of a polymer gelous network structure (Si-O-Si or Si-O-Al). The positively charged surface of the fly ash samples at the working pH according to pH_PZC_ (FA—5.11, FAmod_1_—6.28, FAmod_2_—8.69) prevents electrostatic attraction; thus, REE adsorption occurs predominantly by chemisorption. This conclusion can be confirmed by the enthalpy values, which range from 24.25 to 194.8 kJ/mol, and by fitting the results to a pseudo-second-order kinetic model. The increase in the content of exchangeable sodium and calcium ions in the modified samples contribute to the removal of REE ions by the ion exchange process. By varying the percentage of wood ash added, this impact can potentially be further increased.

The highest adsorption efficiency values (up to 95%) were achieved for scandium on the modified FAmod_2_ material, even in the presence of other ions. The obtained results indicate that the alkali-modified fly ash could potentially be used for the preconcentration of REEs from acidic leachates. The alkaline activation of raw fly ash and fly ash with the addition of wood ash yields effective adsorbent materials for the uptake of REEs from water, and is also an effective way to utilize industrial waste resource. The materials used could be further used as additives for the pavement of roads or in building materials, thereby preventing their environmental impact.

## Figures and Tables

**Table 1:** Chemical composition of the unmodified and modified fly ash samples.

Sample	Weight %								
	C	O	Na	Mg	Al	Si	K	Ca	Fe
FA	1.75	52.97	0.44	1.16	12.59	23.62	1.06	1.48	4.92
FAmod_1_	5.68	45.23	8.17	1.01	8.59	25.28	0.83	1.56	3.65
FAmod_2_	2.34	43.28	8.05	1.06	8.20	27.61	2.47	4.85	2.14

**Table 2:** Kinetic parameters for Gd, Y, and Sc adsorption onto FA, FAmod_1_, and FAmod_2_.

Sample	Analyte	Pseudo-First Order			Pseudo-Second Order			*q_e_*_,exp_, mg g^−1^
		*q_e_*_,cal_ mg g^−1^	*k*_1_, min^−1^	*R* ^2^	*q_e_*_,cal_, mg g^−1^	*k*_2_, g(mg min)^−1^	*R* ^2^	
FA	Gd	0.848	0.124	0.34968	0.908	0.195	0.71598	0.985
	Y	1.156	0.014	0.94526	1.257	0.017	0.95099	1.180
	Sc	1.462	0.045	0.56145	1.601	0.041	0.84735	1.690
FAmod_1_	Gd	4.218	0.032	0.88462	4.645	0.010	0.98574	4.630
	Y	3.956	0.013	0.98328	4.323	0.004	0.92832	3.864
	Sc	3.144	0.066	0.61290	3.380	0.030	0.88727	3.420
FAmod_2_	Gd	4.765	0.030	0.63952	5.105	0.010	0.81121	5.090
	Y	3.907	0.020	0.91162	4.245	0.007	0.93706	4.020
	Sc	4.622	0.040	0.91561	5.058	0.011	0.98867	5.010

**Table 3:** Intraparticle diffusion parameters and correlation coefficients for adsorption of Gd, Y, and Sc onto fly ash samples.

Sample	Analyte	*R*_1_^2^	*k_id,_*_1_, mg/g min^1/2^	*C*_1_, mg/g	*R*_2_^2^	*k_id,_*_2_, mg/g min^1/2^	*C*_2_, mg/g	*R*_3_^2^	*k_id,_*_3_, mg/g min^1/2^	*C*_3_, mg/g
FA	Gd	0.94591	0.0250	0.565	1	0.0030	0.872			
	Y	0.98627	0.0776	0.038	1	0.0047	1.001			
	Sc	0.99479	0.0694	0.592	1	0.0074	1.408			
FAmod_1_	Gd	1	0.5028	0.034	0.97556	0.1905	1.703	1	0.0183	3.934
	Y	0.97649	0.3124	0.037	1	0.0070	3.598			
	Sc	0.94827	0.1892	1.301	0.89001	0.0857	2.174	1	0.0055	3.211
FAmod_2_	Gd	0.91658	0.2897	1.264	1	0.0658	3.907	1	0.0122	4.626
	Y	0.98101	0.2588	0.442	1	0.0095	3.661			
	Sc	0.98578	0.4680	0.452	0.83437	0.1179	3.060	1	0.0123	4.543

**Table 4:** Langmuir and Freundlich parameters for Gd, Y, and Sc adsorption onto FA, FAmod_1_, and FAmod_2_.

Sample	Analyte	Freundlich Isotherm			Langmuir Isotherm		
		*Kf*, mg^1−1/n^L ^1/n^ g^−1^	1/*n*	*R* ^2^	*Q_0_*, mg g^−1^	*b*, dm^3^ mg^−1^	*R* ^2^
FA	Gd	0.034	1.237	0.88012	1.506	7.6 × 10^−5^	0.96348
	Y	0.244	0.597	0.87083	2.631	0.063	0.94216
	Sc	0.116	1.010	0.91900	2.557	0.002	0.94118
FAmod_1_	Gd	2.512	0.277	0.57135	4.771	0.045	0.98281
	Y	1.108	0.506	0.79113	4.431	0.024	0.90815
	Sc	1.104	0.450	0.62850	3.544	5.8 × 10^−4^	0.98037
FAmod_2_	Gd	2.819	0.301	0.58363	5.445	0.105	0.85449
	Y	1.361	0.450	0.79037	4.569	0.041	0.91591
	Sc	3.163	0.221	0.58589	5.136	0.473	0.98707

**Table 5:** Thermodynamic parameters for Gd, Y, and Sc adsorption onto FA, FAmod_1_, and FAmod_2_.

Sample	Analyte	*q*_e_, mg g^−1^			Thermodynamic Parameters				
					∆*G*, kJ mol^−1^			∆*H*, kJ mol^−1^	∆*S,* kJ mol^−1^ K^−1^
		Temperature, K							
		298.15	308.15	318.15	298.15	308.15	318.15		
FA	Y	0.22	0.28	0.57	9.57	8.49	7.42	41.75	0.108
	Sc	0.60	0.72	0.98	6.64	6.07	5.49	24.25	0.059
	Gd	0.024	0.26	0.36	14.13	10.86	7.59	111.6	0.327
FAmod_1_	Y	1.12	2.04	2.12	4.25	2.98	1.71	42.16	0.127
	Sc	2.63	3.90	3.94	−0.30	−5.01	−9.72	140.2	0.471
	Gd	0.89	1.24	1.40	5.28	4.62	3.96	24.97	0.066
FAmod_2_	Y	2.32	3.89	3.92	0.49	−3.83	−7.59	126.1	0.420
	Sc	3.52	3.99	4.00	−4.03	−10.70	−17.37	194.8	0.667
	Gd	3.19	3.50	3.82	−1.57	−4.97	−8.37	99.77	0.340

**Table 6:** Rare earth element adsorption values qe [mg/g] onto different adsorbents according to data in the literature.

Adsorbent	Element	pH	C_0_, mg/dm^3^	q_e_, mg/g	Reference
Amberlite XAD-4 resin impregnated with di (2-ethylhexyl)phosphoric acid	Sc	2.0	1.5	0.034	[45]
Amberlite XAD7HP resin	Sc	3.0	100	23.03	[46]
Bulk graphitic carbon nitride	Gd	6.0	0.06	24	[47]
Fly ash hydrothermally mixed with (3-aminopropyl) triethoxysilane and diethylenetri-aminepentaacetic	Gd	2.0	10	28.9	[48]
Ion adsorption-type rare earth ore	Y	3.0	1000	1.65	[49]
Nanomodified coconut shell activated carbon	Sc	2.0	20	5.8	[50]
Natural bentonite clay	Gd	6.0	20 to 50	96.0	[51]
Pitaya peel biochar	Sc	3.0	30	13.0	[52]
Poly ((sodium styrene sulphonate)-(acrylic acid-polyacrylamide))	Gd	5.5	400	154	[19]
Resin microspheres (SiO_2_-P)	Sc	1.0	15 to 300	8.5	[53]
Titanium dioxide nanoparticles impregnated with Na^+^ cations	Y	7.0	445	33	[18]
Titanium dioxide with surface arsenate groups	Y	9.0	185–4202	26.4	[54]
Alkali-activated fly ash and wood ash (80:20)	Gd	3.5	10	5.090	This study
	Sc			4.020	
	Y			5.010	

## Data Availability

The original contributions presented in this study are included in the article. Further inquiries can be directed to the corresponding author.
